# Large Scale Hydrodynamically Coupled Brownian Dynamics Simulations of Polymer Solutions Flowing through Porous Media

**DOI:** 10.3390/polym14071422

**Published:** 2022-03-31

**Authors:** Vishal Raju Ahuja, Jasper van der Gucht, Wim Briels

**Affiliations:** 1 Shell India Markets Private Limited, Shell Technology Centre Bangalore, Plot No 7, Bangalore Hardware Park, Devanahalli Industrial Park, Mahadeva Kodigehalli, Bengaluru 562149, Karnataka, India; 2 Physical Chemistry and Soft Matter, Wageningen University, Building 124, Stippeneng 4, 6708 WE Wageningen, The Netherlands; jasper.vandergucht@wur.nl; 3 Computational Chemical Physics, Faculty of Science and Technology, University of Twente, P.O. Box 217, 7500 AE Enschede, The Netherlands; 4 MESA+ Institute for Nanotechnology, University of Twente, P.O. Box 217, 7500 AE Enschede, The Netherlands; 5 Forschungszentrum Jülich, IBI 4, D-52425 Jülich, Germany

**Keywords:** Hydrodynamically Coupled Brownian Dynamics, non-Newtonian flow through porous media, polymer flooding, viscoelasticity, shear-thinning polymers, coarse-graining, large scale simulations of polymer solutions

## Abstract

Large scale simulations of polymer flow through porous media provide an important tool for solving problems in enhanced oil recovery, polymer processing and biological applications. In order to include the effects of a wide range of velocity and density fluctuations, we base our work on a coarse-grain particle-based model consisting of polymers following Brownian dynamics coupled to a background fluid flow through momentum conserving interactions. The polymers are represented as Finitely Extensible Non-Linear Elastic (FENE) dumbbells with interactions including slowly decaying transient forces to properly describe dynamic effects of the eliminated degrees of freedom. Model porous media are constructed from arrays of parallel solid beams with circular or square cross-sections, arranged periodically in the plane perpendicular to their axis. No-slip boundary conditions at the solid–fluid interfaces are imposed through interactions with artificial particles embedded within the solid part of the system. We compare the results of our simulations with those of standard Smoothed Particle Hydrodynamics simulations for Newtonian flow through the same porous media. We observe that in all cases the concentration of polymers at steady state is not uniform even though we start the simulations with a uniform polymer concentration, which is indicative of shear-induced cross-flow migration. Furthermore, we see the characteristic flattening of the velocity profile experimentally observed for shear-thinning polymer solutions flowing through channels as opposed to the parabolic Poiseuille flow profile for Newtonian fluids.

## 1. Introduction

We present large scale Brownian dynamics simulations of polymer solutions flowing through complex geometries, with hydrodynamic interactions mediated through explicit solvent. Simulations like these provide an important research tool for various applications.

Polymer flooding for Enhanced Oil Recovery (EOR) is one such application where large scale simulations of polymer solutions through porous media are very important. To understand the need for polymer flooding, it is important to understand the different stages of oil recovery from an oil reservoir. The first stage of oil recovery, known as primary recovery, typically involves the digging of production wells, through which the oil naturally comes out of the reservoir due to the high pressure of the oil in the reservoir and simultaneous rock decompression. This results in the recovery of 5–25% of the Oil Originally In Place (OOIP) depending on the type of oil reservoir. Light oils typically respond well to primary recovery whereas heavy oils and particularly tar sands respond very poorly [1]. The second stage, known as secondary recovery, involves drilling additional wells known as injection wells, through which a displacing fluid such as water or steam is pumped into the reservoir to drive the oil out, which leads to an additional recovery of 5–30% of OOIP depending on the type of oil reservoir. Thus, the most conservative estimate of the oil still remaining in the reservoir after primary and secondary recovery is at least 45% of OOIP and practically in many cases significantly more than 50% of OOIP up to almost 70% of OOIP in some cases [2]. This illustrates the need for a tertiary stage of oil recovery, which is typically done using Enhanced Oil Recovery (EOR) operations such as polymer flooding. The primary reasons for why part of the oil is left behind, even after the second stage, are either that the oil is trapped in the reservoir due to capillary forces or that the displacing fluid is bypassing the oil due to differences in mobility between the two fluids [3,4]. Thus, to recover more oil, one must increase the capillary number and/or reduce the mobility ratio. The addition of polymers to a displacing fluid such as water is thus a well known way to increase its viscosity, which increases the capillary number and diminishes the mobility difference between the displacing fluid and the oil. Furthermore, it also imparts elasticity to the displacing fluid, which leads to further enhancement of the oil recovery. It is this visco-elasticity acquired by the displacing fluid by addition of the polymers that we wish to capture with our model.

Besides its relevance for oil recovery, flow of polymer solutions and polymer gels in complex geometries is interesting in its own right [5]. The interplay between external and internal time and length scales gives rise to phenomena such as cross-flow migration, shear banding [6,7], elastic instabilities and elastic turbulence [8], which are not accessible with small scale simulations in simple geometries. In order to identify the relative importance of shearing and compressing motions, flow through model geometries such as packed beds, periodic arrays of cylinders, and microfluidic devices with pore throats have been investigated [2,9,10,11,12,13,14,15,16].

Traditionally, theoretical work mainly concentrates on constitutive modeling. Early work on polymer systems has been summarized in the books of Doi and Edwards [17] and Graessley [18]. For shear banding, see the review by Olmsted [19], and more recent work by Peterson et al. [20] and references therein. Constitutive models have been used to simulate flow of polymer solutions through porous media by means of finite-element or finite-volume methods [21,22,23,24,25]. These simulations suffer from several problems that are difficult to bypass. Firstly, instabilities usually must be investigated through a perturbative stability analysis on top of some homogeneous flow, or must be imposed in the flow. Secondly, models and calculations become rather involved when concentration gradients are needed to explain the experimental findings. Thirdly, numerical grids become rather dense near sharp elements in the boundaries of the system, leading to a significant increase in the computational effort. The first two of these problems do not occur with particle based simulations, since they automatically include fluctuations of flow gradients and stresses and naturally allow for concentration gradients. Furthermore, problems near sharp boundaries are less prominent with particle based simulations than with constitutive field modeling. However, explicitly simulating particles severely enhances computational efforts and therefore coarse-graining is essential to enable application to systems with large time and length scales. As a result, the models to be used must be highly coarse grained, representing individual polymers by as few degrees of freedom as possible. Of course, computationally cheaper methods exist to bypass some of the problems mentioned above, of which Lattice–Boltzmann simulations are the most prominent [26,27]. This method is designed to properly include Newtonian hydrodynamics, but it is in general very difficult to include non-Newtonian effects into the model without resorting to constitutive equations such as a power law or the Carreau model [28]. We therefore opt for a particle based model, where it is easily possible to incorporate non-Newtonian effects into a self-developing flow arising directly out of molecular interactions. To motivate the particular model that we have chosen to simulate, we quickly review some of the history of coarse grained polymer models.

The science of eliminating ‘irrelevant’ degrees of freedom from particle based simulations is called coarse graining. As a simple example, we mention the use of united atoms to simulate –CH, –${\mathrm{CH}}_{2}$ and –${\mathrm{CH}}_{3}$ groups as (different) single particles in early molecular dynamics simulations. In an effort to simulate properties of realistic, as opposed to generic, polymer systems, united atom coarse graining was extended to larger units such as aromatic and even larger groups [29,30]. For applications in biophysical systems a highly successful course grain force field has been developed called MARTINI [31,32]. Around the same time, it was realized that lumping together even larger, flexible groups of atoms would ask for stochastic simulation methods [33], and in particular for non-Markovian stochastic simulations [34]. The latter field has developed quickly and has been reviewed recently by Klippenstein et al. [35]. On the more generic side of models capturing the chain character of polymers but leaving out chemical information, we mention the groundbreaking Kremer–Grest molecular dynamics model, [36] the blob model of Padding and Briels [37,38] and the slip-link model of Masubuchi et al. [39,40].

All particle based models mentioned so far are too detailed to serve our purpose of performing large scale simulations of flow through large channels. We will have to resort to models in which every polymer is represented by one or two particles or blobs. Representing large but rather low density structures, such particles will severely overlap, resulting in every polymer having many neighbors within its radius of gyration. With the correct potential of mean force, i.e., the free energy of all eliminated degrees of freedom for the given configuration of the coarse degrees of freedom, the thermodynamics of such systems can still be simulated with great accuracy, provided the potential of mean force at hand can be represented in a tractable way. The dynamics of such particles, however, will not be captured with any accuracy at all. The reason for this is that the eliminated degrees of freedom in an all-atom simulation, as a result of the high degree of entanglement of the overlapping particles, would have given rise to a bunch of slow modes on a wide range of time and length scales, which we generically call disentangling. Tube models and corresponding disengagements as presented in the book of Doi and Edwards [17] describe the ‘microscopic’ dynamics of the degrees of freedom that we have eliminated. They slow down the dynamics of the coarse degrees of freedom much more than can possibly be described by the potential of mean force. We must therefore explicitly re-introduce the effects of disentangling processes by hand. We accomplish this using the responsive particle dynamics model (RaPiD) of van den Noort et al. [41] through the introduction of structural degrees of freedom with every pair of polymers, whose deviation from equilibrium gives rise to transient forces [41,42] acting on the particles. From the point of view of dynamics, these forces impose non-Markovian friction and random forces on the particles. In other words, they essentially provide memory to the forces that act on the particles. In order to include elastic forces, we represent every polymer by a dumbbell molecule containing two blobs connected by a Finitely Extensible Non-linear Elastic (FENE) spring [43].

A second aspect that is absent in most coarse grain polymer models but is important in the flow of low density polymer solutions is the possibility of hydrodynamic interactions. This is the transport of momentum resulting from movements of one polymer mediated through the solvent and imparted to another polymer. In this paper, we include the presence of the solvent through the simulation of explicit fluid blobs as in Smoothed Particle Hydrodynamics (SPH) [44]. In order to guarantee correct hydrodynamic behavior, we must couple the polymer and fluid blobs in such a way that local momentum conservation applies. For this reason, we use our recently published two-way coupling technique ‘Hydrodynamically Coupled Brownian Dynamics’ (HCBD) described in Ahuja et al. [45]. For applying the no-slip condition, we use artificial particles embedded within the solid beams as we have done in our previous work [46]. From preliminary simulations with this model we have found that it exhibits cross-flow migration under shear as well as characteristic flattening of the Poiseuille flow profile observed for polymer solutions.

Thus, in this paper, we give a proof of principle that our Hydrodynamically Coupled Brownian Dynamics (HCBD) model can be used to perform large scale simulations of polymer solution flow through channels with sharp boundary elements, with only limited computational effort. To this end, we study the flow of such systems through model porous media, which are constructed using periodic arrays of solid beams arranged on a square grid, the plane of which is perpendicular to the axes of the beams. We study the flow of our model polymer solution across two different types of beams—one with circular cross-sections which we call cylindrical beams and another one having square cross-sections which we call cuboidal beams to simulate both the curved and sharp interfaces found in naturally occurring porous media. Furthermore, we study two extreme angles of attack—one parallel to one of the Cartesian directions of the plane of the square grid of beams and another at an equal angle (45°) from either Cartesian directions of the same plane. Thus, we study four different cases—two model porous media geometries and two flow directions. We also compare the results with the flow of pure Newtonian solvent, which we have simulated using standard SPH simulations.

Before embarking on a description of our model, we cite some other work in this field [47], as well as bring some journals to the attention of the readers that are less well known in the traditional polymer physics community [48,49,50,51,52].

## 2. Method

In this section, we present the equations of motion of all particles present in our simulation boxes. These encompass the blobs that constitute a polymer, and those that make up the SPH based solvent.

### 2.1. Equation of Motion for the Polymer Blobs

The position $r_{a}$, shorthand for $r_{a}\left(t\right)$, of the center-of-mass of any given blob (or lobe) *a* at time *t* is updated according to:

(1) $$dr_{a}=v\left(r_{a}\right)dt+\left(\frac{F_{a}}{\xi_{a}}\right)dt+k_{B}T\frac{\partial }{\partial r_{a}}\left(\frac{1}{\xi_{a}}\right)dt+dW_{a}^{r}.$$

Here, dt is the time-step, $dr_{a}=r_{a}\left(t+dt\right)-r_{a}\left(t\right)$, and $F_{a}=F_{a}\left(t\right)$ is the driving force acting on blob *a* as a result of the interaction with other such blobs, in addition to any force field that may have been applied. $\xi_{a}=\xi \left(r_{a}\left(t\right)\right)$ is the friction coefficient at the position of polymer blob *a*. The third term on the right hand side of Equation (1) is a drift term accounting for the spatial variation of the friction coefficient, needed to guarantee the correct canonical equilibrium distribution in the quiescent state. For the sake of simplicity, we have assumed a constant friction coefficient, thereby rendering the third term equal to zero. The last term on the right hand side of Equation (1), i.e., $dW_{a}^{r}=dW_{a}^{r}\left(t\right)$, is a random displacement typical of Brownian dynamics simulations. This random displacement is uncorrelated in time and has a magnitude that is calculated in accordance with the fluctuation dissipation theorem, satisfying:

(2) $$\langle dW_{a}^{r}dW_{b}^{r}\rangle =2k_{B}T\left(\frac{dt}{\xi_{a}}\right)\delta_{ab}I.$$

Hereafter in this paper, we will not include the *t* in our notation, tacitly assuming that it is implicitly present. Furthermore, $v(r_{a})$ in Equation (1) is the background fluid velocity at the position of blob *a*. It is calculated as an interpolation of the velocities $v_{i}$ of the fluid blobs in the vicinity of $r_{a}$ using an appropriately normalized weight function $w^{f}\left(r\right)$ as shown below:

(3) $$v\left(r_{a}\right)=\sum_{i=1}^{N_{f}}\frac{w^{f}\left(r_{ai}\right)}{n_{i}^{f}}v_{i},$$

where *i* runs over the $N_{f}$ fluid blobs, and $n_{i}^{f}$ is the local number density of fluid blobs calculated as $\sum_{j=1}^{N_{f}}w^{f}\left(r_{ij}\right)$.

Based on the RaPiD polymer model for FENE dumbells, the force $F_{a}$ can be expressed as a sum of three terms as shown below:

(4) $$F_{a}=-\frac{\partial }{\partial r_{a}}\left[\Phi_{c}+\Phi_{t}+\Phi_{f}\right],$$

where $\Phi_{c}$ is the so-called ‘conservative’ potential, $\Phi_{t}$ is the so-called ‘transient’ potential and $\Phi_{f}$ is the FENE potential, all of which shall be defined and described in Section 3.

### 2.2. Equation of Motion for the Fluid Blobs

Consider a fluid blob *i* naturally moving with the background flow field at its position of its center-of-mass. The position of its center-of-mass is thus updated using:

(5) $$dr_{i}=v_{i}dt.$$

For calculating the flow field, we discretize the Navier–Stokes equation as in SPH and introduce an additional term coupling the fluid motion to the polymer motion as part of the HCBD two-way coupling technique [45]. Thus, we arrive at the following equation that we have used in our simulations to update the velocities of any given fluid blob *i*:

(6) $$\begin{array}{ccc} dv_{i}= & - & \frac{dt}{m}\left[\sum_{j=1}^{N_{f}}\left(\frac{P_{i}}{{\left(n_{i}^{f}\right)}^{2}}+\frac{P_{j}}{{\left(n_{j}^{f}\right)}^{2}}\right)\frac{dw^{f}}{dr}\left(r_{ij}\right)\frac{r_{ij}}{r_{ij}}+\sum_{j=1}^{N_{f}}f_{ij}v_{ij}\right]+g_{i}dt\\ & + & \frac{dt}{m}\left[\sum_{a=1}^{N_{b}}\frac{w^{f}\left(r_{ai}\right)}{n_{i}^{f}}F_{a}\right]+\sum_{j=1}^{N_{f}}dW_{ij}^{v}, \end{array}$$

where *m* is the mass of the fluid blob *i*, $N_{f}$ is the number of fluid blobs, $P_{i}$ is the pressure at the position of the fluid blob *i* and $n_{i}^{f}$ is the local number density of fluid blobs. $f_{ij}=f\left(r_{ij}\right)$, is a symmetric function defined as follows:

(7) $$f\left(r_{ij}\right)=\left\{\begin{array}{cc} -\left(\frac{2\eta }{n_{i}^{f}n_{j}^{f}}\right)\frac{1}{r_{ij}}\frac{dw^{f}}{dr}\left(r_{ij}\right)\; & forr_{ij}\leq R_{c}\\ 0 & forr_{ij}\geq R_{c}, \end{array}\right.$$

with η being the solvent viscosity. Moreover, $v_{ij}$ is the velocity of fluid blob *i* relative to that of *j* and $g_{i}$ is the acceleration due to body forces. The pairwise velocity fluctuation terms are uncorrelated in time and anti-symmetric manner, in the sense that $dW_{ij}^{v}=-dW_{ji}^{v}$, in order to conserve momentum. The exact properties of the momentum fluctuations are taken in such a way that the steady state probability distribution of the positions and velocities of the fluid in a quiescent state yields the expected canonical equilibrium distribution. Thus, we have:

(8) $$\begin{array}{ccc} \langle dW_{ij}^{v}dW_{ij}^{v}\rangle & = & \left(\frac{2k_{B}T}{m}\right)\left(\frac{dt}{m}\right)f_{ij}I, \end{array}$$

(9) $$\begin{array}{ccc} \langle dW_{ik}^{v}dW_{jl}^{v}\rangle & = & 0\;\;\;(ik\neq jl\wedge ik\neq lj). \end{array}$$

For details of the derivation, see our previous work on these matters [45,53].

The third term in Equation (6) provides the coupling of the fluid motion to that of the polymer. Here, $N_{b}$ is the number of polymer blobs (which is twice the number of polymers $N_{p}$ as each polymer is represented by two blobs). In order to have correct hydrodynamic behavior, it is important that the coupling between the polymer and the fluid motion locally conserves momentum. For a proof of this fact, we refer to our previous work [45].

In cases of appreciable flow-rates at the current scale of the simulation, the random contributions to the fluid forces may be neglected, as we do in this paper. However, with low velocities and for simulations at smaller scales, one must add the additional fluctuation terms as mentioned in the equation.

### 2.3. Solutions of the Equations of Motion

The first order Brownian dynamics equations for the positions of the polymers were updated according to a first order Euler equation for the systematic forces, i.e., literally as given in Equation (1), with the random contribution calculated as follows:

(10) $$dW_{a}^{r}=\sqrt{\frac{2k_{B}T}{\xi }\Delta t}\left(\begin{array}{c} G_{x}\\ G_{y}\\ G_{z} \end{array}\right),$$

where Δt is the time-step and $G_{\alpha }$ are Gaussian random numbers with mean zero and unit variance, i.e., with $<G_{\alpha }>=0$ and $<G_{\alpha }G_{\beta }>=\delta_{\alpha ,\beta }$.

The second order dynamics equations for the motion of the fluid blobs were integrated with the leap-frog algorithm for the conservative forces and frictional forces augmented with random contributions to velocity and positions of the fluid blobs being calculated as follows

(11) $$\begin{array}{ccc} dW_{ij}^{v} & = & \frac{\sqrt{2k_{B}Tf_{ij}\Delta t}}{m}\;G_{ij}\left(\frac{r_{ij}}{r_{ij}}\right), \end{array}$$

(12) $$\begin{array}{ccc} dr_{i}^{ran} & = & \sum_{j=1}^{N_{f}}dW_{ij}^{v}dt \end{array}$$

in that order. Here, it is understood that $G_{ij}=G_{ji}$ and for the rest, these random numbers have the same properties as the Gaussian random numbers defined earlier as $G_{\alpha }$.

## 3. Force Fields

In this section, we define the potentials from which the forces on the polymers are derived and the equation of state that enters as a force in the equations of motion of the fluid blobs.

### 3.1. The Conservative Potentials

There are two force fields from which conservative forces are derived, i.e., $\Phi_{c}$ and $\Phi_{f}$. As already mentioned in the Introduction, the interaction potential between coarse grain polymers is actually the free energy of all eliminated degrees of freedom for the given configuration of the coarse degrees of freedom. In order to define $\Phi_{c}$ we treat the polymer as a single entity and calculate everything needed from the center-of-mass of the polymer instead of the two beads or lobes of the polymer dumbbell. We have used the Flory–Huggins potential, which has been introduced before for simulations of polymer solutions [45,54,55,56], and which is defined as follows:

(13) $$\Phi_{c}=pk_{B}T\sum_{a=1}^{N_{p}}\left[\left(\frac{1-\phi_{a}}{\phi_{a}}\right)\ln\left(1-\phi_{a}\right)-\chi \phi_{a}\right].$$

Here, $N_{p}$ is the total number of polymers in the solution, *p* is the number of Kuhn segments per polymer, χ is the solvent interaction parameter and $\phi_{a}$ is the local volume fraction of polymer blobs in the neighborhood of polymer blob *a* at time *t* calculated as:

(14) $$\phi_{a}=\frac{n_{a}^{p}}{n_{max}^{p}}.$$

In this equation, $n_{max}^{p}$ is the maximum number density of polymers that the system is allowed to reach, i.e., the melt density, and $n_{a}^{p}$ is the local number density at the position of the center-of-mass of polymer blob *a* calculated as:

(15) $$n_{a}^{p}=\sum_{b=1}^{N_{p}}w^{p}\left(r_{ab}\right),$$

where $r_{ab}$ is the distance between the centers-of-mass of polymers *a* and *b* at time *t* and $w^{p}\left(r\right)$ is a normalized weight function with a cut-off $r_{c}$. The superscript *p* in $w^{p}\left(r\right)$ indicates that this is the weight function used for the polymer blobs.

The interaction between two blobs within one polymer is described by the FENE potential $\Phi_{f}$ describing a finitely extensible nonlinear elastic spring, which is given by:

(16) $$\Phi_{f}=-\frac{1}{2}kr_{0}^{2}\sum_{a=1}^{N_{p}}\ln\left[1-{\left(\frac{r_{a}}{r_{0}}\right)}^{2}\right],$$

where $r_{a}$ is the separation between the two lobes of the polymer dumbbell *a*, *k* is the spring constant, and $r_{0}$ is the maximum allowed deformation of the spring.

### 3.2. The Transient Potential

We have used the transient potential of the RaPiD model [41,42] to incorporate memory effects into the simulation model. This potential, which essentially takes into account the history of the interacting polymer blobs by keeping track of additional dynamic variables [57], is given by:

(17) $$\Phi_{t}=\frac{1}{2}\alpha \sum_{a,b=1}^{N_{b}}{\left(\lambda_{ab}-\lambda_{ab}^{eq}\right)}^{2}.$$

$\Phi_{t}$ the transient potential, α is a parameter associated with the strength of the interactions or in other words the penalty for the deviation of the dynamic variable $\lambda_{ab}=\lambda_{ab}\left(t\right)$ from its equilibrium value $\lambda_{ab}^{eq}$, shorthand for $\lambda^{eq}\left(r_{ab}\right)$. We use here the following form for $\lambda^{eq}\left(r_{ab}\right)$ that has been used earlier in the literature [54,55,56]:

(18) $$\lambda^{eq}\left(r_{ab}\right)=\left\{\begin{array}{cc} {\left(1-\frac{r_{ab}}{r_{c}}\right)}^{2}\; & \mathrm{for}\;r_{ab}\leq r_{c}\\ 0 & \mathrm{for}\;r_{ab}\geq r_{c}. \end{array}\right.$$

The variable $\lambda_{ab}$ is a dimensionless variable representing the degree of intermixing of the polymers *a* and *b*, which evolves over time according to the following first order stochastic differential equation:

(19) $$d\lambda_{ab}=-\left(\lambda_{ab}-\lambda_{ab}^{eq}\right)\frac{dt}{\tau }+dW_{ab}^{\lambda },$$

where τ is the relaxation time and $W_{ab}^{\lambda }$ is a Wiener process with time-uncorrelated increments satisfying:

(20) $$\langle dW_{ab}^{\lambda }dW_{ab}^{\lambda }\rangle =\left(\frac{2k_{B}T}{\alpha }\right)\left(\frac{dt}{\tau }\right)I.$$

In a non-flowing system, $\lambda_{ab}$ will harmonically fluctuate around its equilibrium value.

### 3.3. The Equation of State

For the pressure, acting as a force in the dynamical updates of the velocities of the fluid blobs, we use the commonly used pseudo-incompressible equation of state [58,59] given by:

(21) $$P_{i}=P_{0}\left[{\left(\frac{n_{i}^{f}}{{\bar{n}}^{f}}\right)}^{7}-1\right],$$

where $P_{0}$ is chosen such that the velocity of sound in the simulation is sufficiently large in order that the density fluctuations are sufficiently small, which results in a fluid that resembles an incompressible fluid.

## 4. Interaction of the Fluid with the Solid

For the correct solid–fluid interaction, there are two important conditions that need to be met—no penetration of fluid into the solid and a no-slip boundary condition at the solid–fluid interface. For this purpose, we explicitly embed artificial particles uniformly distributed in the solid regions with the same density as the real fluid blobs as in our previous work [46]. These artificial particles interact with the real fluid blobs in a couple of ways. Firstly, they contribute to the density of the fluid blobs in their vicinity and consequently the pressure as well. Furthermore, the density and pressure is also calculated for the artificial particles in the vicinity of the real fluid blobs. This ensures that the pressure inside the solid is sufficient to prevent the fluid from entering the solid. Secondly, for ensuring the no-slip boundary condition at the surface of the solid, we apply the Morris boundary conditions [60], which is applicable for plane as well as curved boundaries. We also use these artificial particles to calculate densities in the Flory–Huggins potential for the polymers, albeit with a higher weight. The weight assigned to artificial particles is higher than regular polymer blobs by a ratio of the density of the polymers to the density of the artificial particles. This is to compensate for the difference in the density of these artificial particles and the polymers. This ensures that polymers do not artificially accumulate near the solid interface owing to lower concentration in a hypothetical sphere drawn around the center of these polymer blobs at the interface. Moreover, if still any polymer blob enters the solid due to a random displacement, we explicitly forbid that move, thereby preventing the polymer blob from entering the solid due to a random displacement.

## 5. Weights and System Parameters

For the polymer blobs, we have used a normalized weight function that has been used earlier in the literature, where the polymer model RaPiD has been employed [45,54,55,56]. It given by $w^{p}\left(r\right)$:

(22) $$w^{p}\left(r\right)=\left\{\begin{array}{cc} \frac{15}{2\pi (r_{c}^{5}-\sigma^{5})}\left(r_{c}-\sigma \right)\left(r_{c}+\sigma -2r\right)\; & \mathrm{for}\;r\leq \sigma \\ \frac{15}{2\pi (r_{c}^{5}-\sigma^{5})}{\left(r-r_{c}\right)}^{2}\; & \mathrm{for}\;\sigma \leq r\leq r_{c}\\ 0 & \mathrm{for}\;r\geq r_{c}, \end{array}\right.$$

where the cut-off $r_{c}$ is chosen as 2.5σ.

For the fluid blobs, we have chosen the normalized $M_{4}$ kernel commonly used in SPH [44] as the weight function. It is given by $w^{f}\left(r\right)$:

(23) $$w^{f}\left(r\right)=\left\{\begin{array}{cc} \frac{1}{4\pi h^{3}}\left[{\left(2-\frac{r}{h}\right)}^{3}-4{\left(1-\frac{r}{h}\right)}^{3}\right]\; & \mathrm{for}\;r\leq h\\ \frac{1}{4\pi h^{3}}{\left(2-\frac{r}{h}\right)}^{3} & \mathrm{for}\;h\leq r\leq 2h\\ 0 & \mathrm{for}\;r\geq 2h, \end{array}\right.$$

where *h* is what is commonly referred to as the support of the weight function, which we have chosen as h=2σ for our simulations such that the cutoff radius $R_{c}=2h$ for the fluid blobs is larger than the cut-off radius $r_{c}=2.5\sigma$ chosen for the polymer blobs. This is done intentionally because the weight function $w^{f}\left(r\right)$ must be able to accurately estimate the second order derivatives occurring in the equation of motion for the fluid blobs. Following the same logic, it immediately follows that for the polymer–fluid interactions, as we do not need to calculate any gradients, we can use the weight function $w^{p}\left(r\right)$ with a smaller cut-off $r_{c}$ instead of $w^{f}\left(r\right)$ with a larger cut-off $R_{c}$ for computational efficiency. Moreover, we must emphasize that we have chosen the resolution for the fluid ${\bar{n}}^{f}$ such that there are on average 8 particles within a sphere of radius *h* so that there are about 64 neighbouring fluid blobs on average, which is sufficiently higher than the minimum required number for this weight function for 3-D SPH simulations [61]. The values of the system parameters have been summarized in Table 1.

It is pertinent to note that the relative resolution between the polymer and the fluid, lets call it R=σ/h, which we have chosen as 1/2 for the work presented in this paper, is actually a very important parameter. In principle, one should choose a high enough value for the relative resolution *R* in order to accurately mimic the draining of the solvent through the polymers. Nevertheless, in practice, if one chooses a very large value for *R*, a direct consequence would be that the fluid blobs have a really small mass and hence a much smaller time-step would be required, which would drastically affect the efficiency of the method. Furthermore, in case of studying flow through a particular geometry, naturally a much larger number of fluid blobs would be required if the same length scale of the geometry is to be studied with smaller blobs. Thus, a very high resolution leads to computational problems. On the other hand, if the value is chosen to be too small, then there would be a large number of polymers per fluid blob. This leads to a conceptual problem because then one would have to capture the fluid flow field gradient within each fluid blob and transmit it to the polymers within the blob, which will need a modification of the equation of motion for the polymers adding an extra term accounting for the gradient of the fluid flow field within the fluid blob. Thus, we have chosen the sizes of the polymer blobs not very different from that of the fluid blobs.

The physical properties of the fluid, i.e., the density and viscosity, have been chosen to be consistent with those of water. The value of $P_{0}$ has been chosen high enough to ensure small density fluctuations and a large enough velocity of sound $c_{s}$ in the simulation, which can be calculated as:

(24) $$c_{s}=\sqrt{\frac{\partial P}{\partial \rho }}=\sqrt{\frac{7P_{0}}{\rho }}.$$

We have ensured that the velocities that we encounter in the simulations performed in this study are much smaller than this velocity of sound in our simulation. For the flow through a cylindrical porous medium in the diagonal direction, a ten times higher value of $P_{0}$ than mentioned in Table 1 has been selected to ensure that the fluid does not break apart downstream of the cylindrical beam, which was found to occur at lower $P_{0}$ values.

The solute particles can be thought of as very large polymers or micro-gel particles with a radius of 5 μm and a relaxation time of 1 s. These may even qualify as small clusters of polymers as may occur when poly-electrolytes like the ones used in enhanced oil recovery, at particular salt concentrations and pH conditions, form salt-bridges among each other.

## 6. Results and Discussion

In this section, we present the results of flow simulations of polymer solutions through two types of channels in porous media. We will mention many cases where polymer concentrations show large gradients as a result of shear banding type of flow instabilities. It is important to mention that in all these cases the overall density of polymers and fluid was always nearly constant throughout the whole part of the systems composed of channels.

### 6.1. Flow through Cylindrical Porous Media

In this sub-section, we present results of flow simulations of our model polymer solution through cylindrical porous media. By cylindrical porous media, we mean that our model porous media consist of solid cylindrical beams with axes along the *z* direction arranged on a square grid on the x-y plane along the Cartesian axes. The radius of each of the cylindrical beams is 40 σ, i.e., 200 microns and the perpendicular distance between the axes of the cylinders in *x* as well as *y* direction is 90 σ, i.e., 450 microns. This leads to a porous medium with a porosity of 0.38. In all the simulations that we have presented in this paper, we have maintained the same porosity. The depth of the box, i.e., its length in *z* direction is 90 microns. The simulation runs for the flow of polymer solutions are started with a steady state profile of the fluid and polymers are added randomly in a uniform manner throughout the box except in the solid region and then the run is continued till steady state is achieved.

#### 6.1.1. Pressure Drop in the Positive *x* Direction

**Figure 1:**
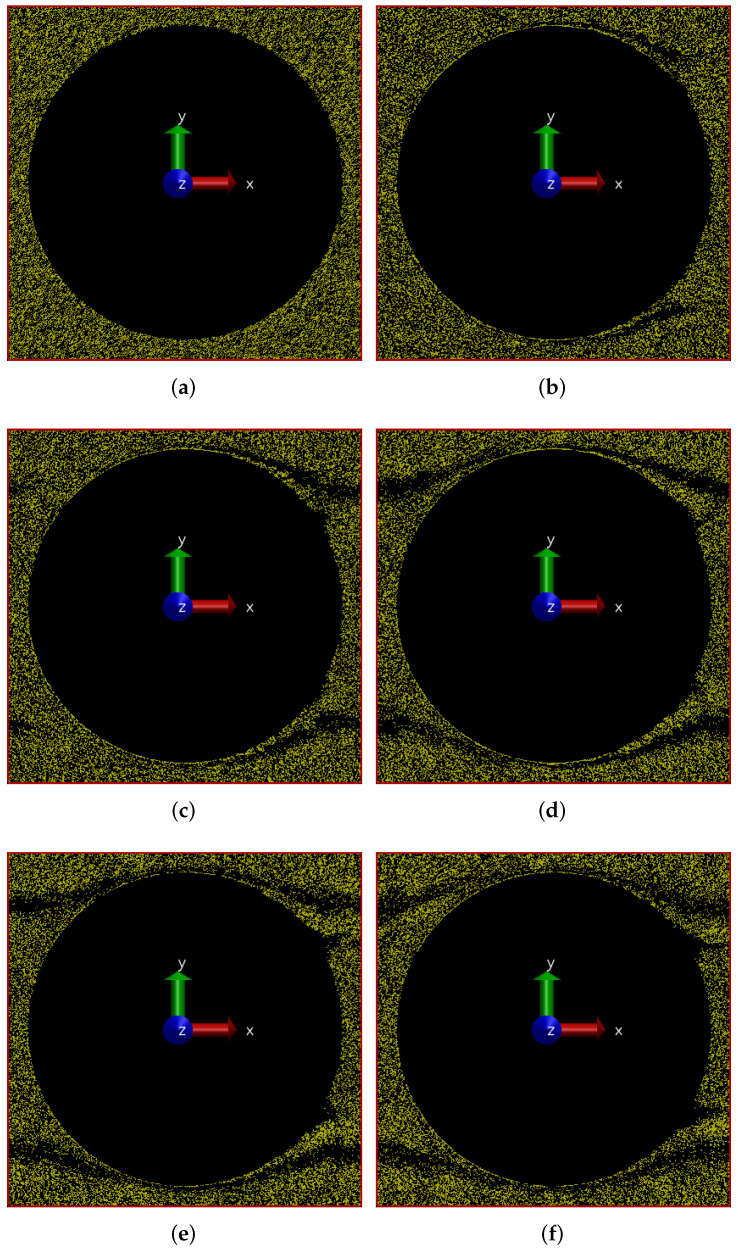
Snapshots of simulation (in beam-view) at different times showing development of polymer positions over time for flow through a cylindrical porous medium with pressure applied in the positive *x*-direction. (**a**) t = 0 s; (**b**) t = 25 s; (**c**) t = 50 s; (**d**) t = 100 s; (**e**) t = 150 s; (**f**) t = 200 s.

We now present results of simulations of flow driven by an applied pressure drop in the positive *x* direction which is effected in the simulation through a body force which produces an acceleration of 0.1 m/$s^{2}$. In Figure 1, we show the positions of polymers in our simulation at different times.

In this figure, we have shown the simulation box in what we call the ‘beam-view’, where the solid region is present at the center of the simulation box and the fluid flows around it. We have also shown the directions of the axes to clarify at the outset what we mean when we refer to *x*, *y* and *z* directions. As can be seen in Figure 1a, the polymers are initially uniformly and randomly distributed throughout the box except the solid region which is the cylinder in the center of the box seen as a circle in this 2-D view. As time progresses, the polymers start forming a pattern which evolves over time and finally settles in a more-or-less steady-state pattern at 200 s as shown in Figure 1f. We clearly see the result of cross-flow migration, which initially is more prominent where the flow is away from the cylinder, i.e., in the downstream area, than in the area where the flow is into the cylinder. It is interesting to note that even in the steady state there are small differences between distributions downstream and upstream. It seems like downstream a second low density streak emerging from the cylinder has developed, while upstream this is not the case. For a more clear visualization of the evolution of polymer positions, please refer to the animations of the simulations provided in the supplementary material.

**Figure 2:**
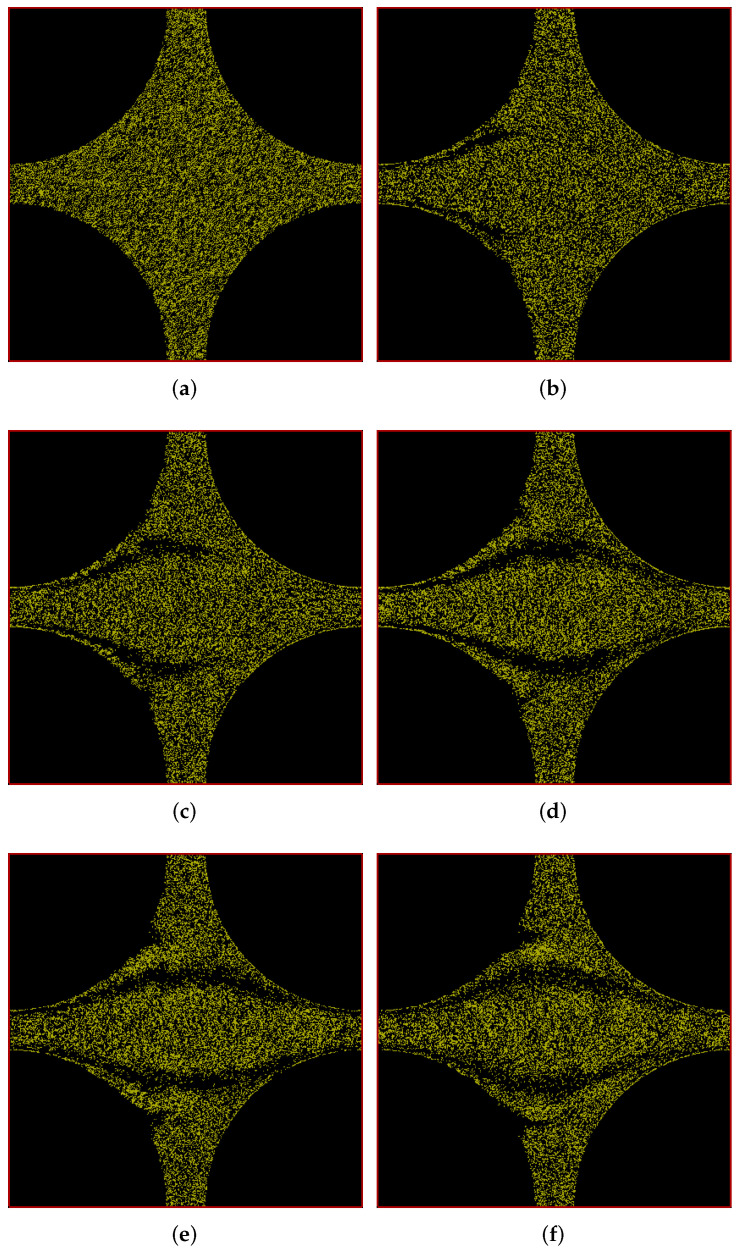
Snapshots of simulation (in porous-media-view) at different times showing development of polymer positions over time for flow through a cylindrical porous medium with pressure applied in the positive *x*-direction. (**a**) t = 0 s; (**b**) t = 25 s; (**c**) t = 50 s; (**d**) t = 100 s; (**e**) t = 150 s; (**f**) t = 200 s.

Since the simulation box is periodic in nature, we can also alternatively represent the results shown above in a different representation which we call the ‘porous-media-view’ as shown in Figure 2. In this view, the pore appears centrally and the solid region is at the corners. Note that we no longer show the axes in this figure and hereafter in all such figures as the notation has been already clarified in Figure 1. From now on, we shall present all the results in this porous-media-view as it provides a better visual impression of the porous media to the reader.

**Figure 3:**
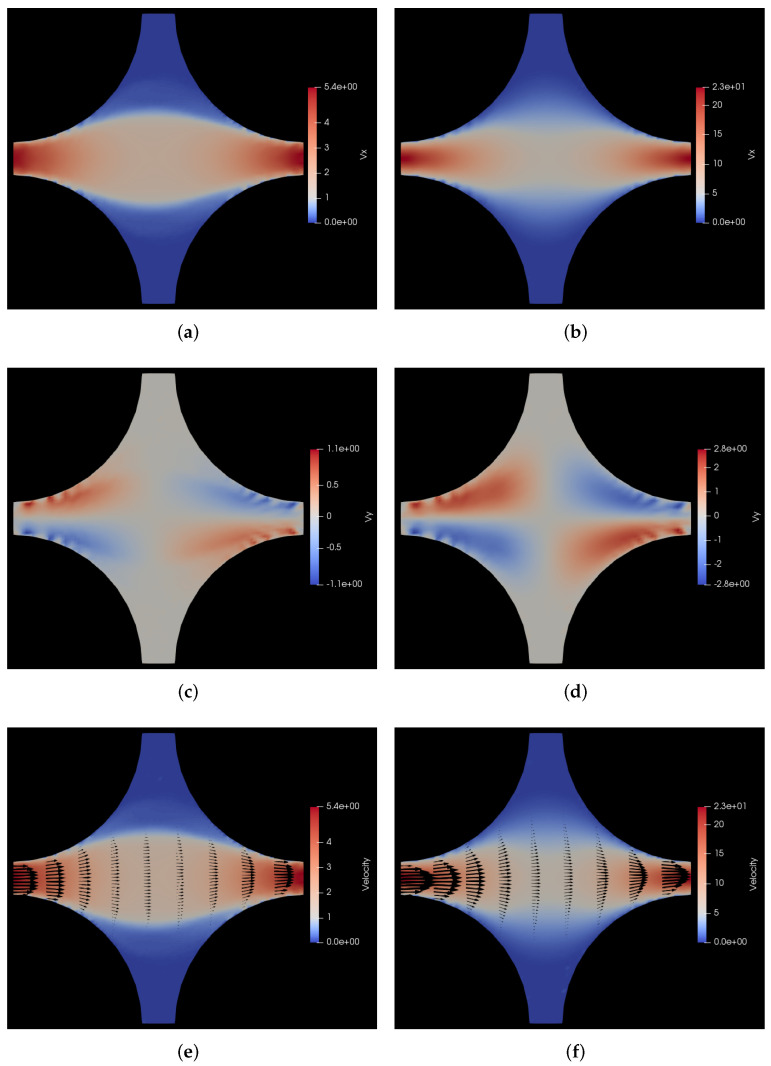
Comparison of velocity profiles for flow of polymer solution with the flow of solvent through a cylindrical porous medium with pressure applied in the positive *x* direction. (**a**) $v_{x}$ for flow of polymer solution. Here, $v_{x}$ varies from 0 to 5.4 σ/s, i.e., 0 to 27 μm/s; (**b**) $v_{x}$ for flow of solvent. Here, $v_{x}$ varies from 0 to 23 σ/s, i.e., 0 to 115 μm/s; (**c**) $v_{y}$ for flow of polymer solution. Here, $v_{y}$ varies from −1.1 to 1.1 σ/s, i.e., −5.5 to 5.5 μm/s; (**d**) $v_{y}$ for flow of solvent. Here, $v_{y}$ varies from −2.8 to 2.8 σ/s, i.e., −14 to 14 μm/s; (**e**) *v* for flow of polymer solution. Here, *v* varies from 0 to 5.4 σ/s, i.e., 0 to 27 μm/s; (**f**) *v* for flow of solvent. Here, *v* varies from 0 to 23 σ/s, i.e., 0 to 115 μm/s.

In Figure 3, we have shown a comparison of the steady state velocity profiles of the flow of polymer solution through the porous medium with the flow of the solvent through the same medium for the same pressure drop. On the left hand side panels of such figures, we show the polymer solution flow profile and on the right hand side panels, we show the solvent flow profile. In fact, the polymer simulations are initiated with the fluid steady state profiles shown in the right panels. Note that the velocities in the legends of all such velocity heat map figures in this paper are shown in internal units of σ/s, i.e., the velocities in S.I. units [m/s] can be obtained by multiplying the velocities in internal units with 5 × $10^{-6}$, as is shown in the captions.

For a better understanding, we have shown not just the overall velocity but also its 2 components, i.e., $v_{x}$ and $v_{y}$ in Figure 3a,c, respectively, for the polymer solution flow and similarly in Figure 3b,d for the solvent flow. Furthermore, in Figure 3e,f, we have shown the velocity vectors superimposed on the velocity heat map for the polymer solution flow and solvent flow, respectively. From these two sub-figures, it can be seen that the maximum velocity *v* in the polymer solution flow is almost 4.6 times lower than the same for the solvent flow through the same porous medium for the same pressure drop. This is due to increased viscosity of the solution due to the addition of polymers to the solvent. Furthermore, the viscosity of the polymer solution is not constant but rather shear-thinning in nature, which leads to the characteristic flattening of the velocity profile in the channels that is clearly visible for the polymer solution flow vis-à-vis the parabolic Poiseuille flow profile for the Newtonian solvent flow.

Furthermore, on comparison of Figure 2 and Figure 3e, we can see that the polymer concentration is reduced in the zones separating the regions of strong primary flow with the regions of near stagnant flow, which indicates shear induced cross-flow migration. This shows that the concentration of the polymers in the flow through the porous medium is not uniform but rather related to the flow through the particular geometry. This level of information is difficult to get with a continuum-level CFD simulation and it demonstrates the importance of particle-based simulations in linking the macroscopic flow to the microscopic molecular level interactions and positions of particles involved in the flow. Needless to say, a more detailed description of the molecules can provide even more information, but to go to a finer level of coarse-graining becomes very expensive if one has to simulate the flow of even several tens of thousands of polymers flowing through a model periodic porous medium like we show here.

#### 6.1.2. Pressure Drop along the Positive *x*-*y* Diagonal

We now present results of flow simulations through our cylindrical porous medium driven by an applied pressure drop in the diagonal direction 45 degrees to the positive *x* and *y* directions. This is effected in the simulation through body forces along the positive *x* and *y* directions, which together produces an acceleration of 0.1 m/$s^{2}$ along the diagonal direction.

**Figure 4:**
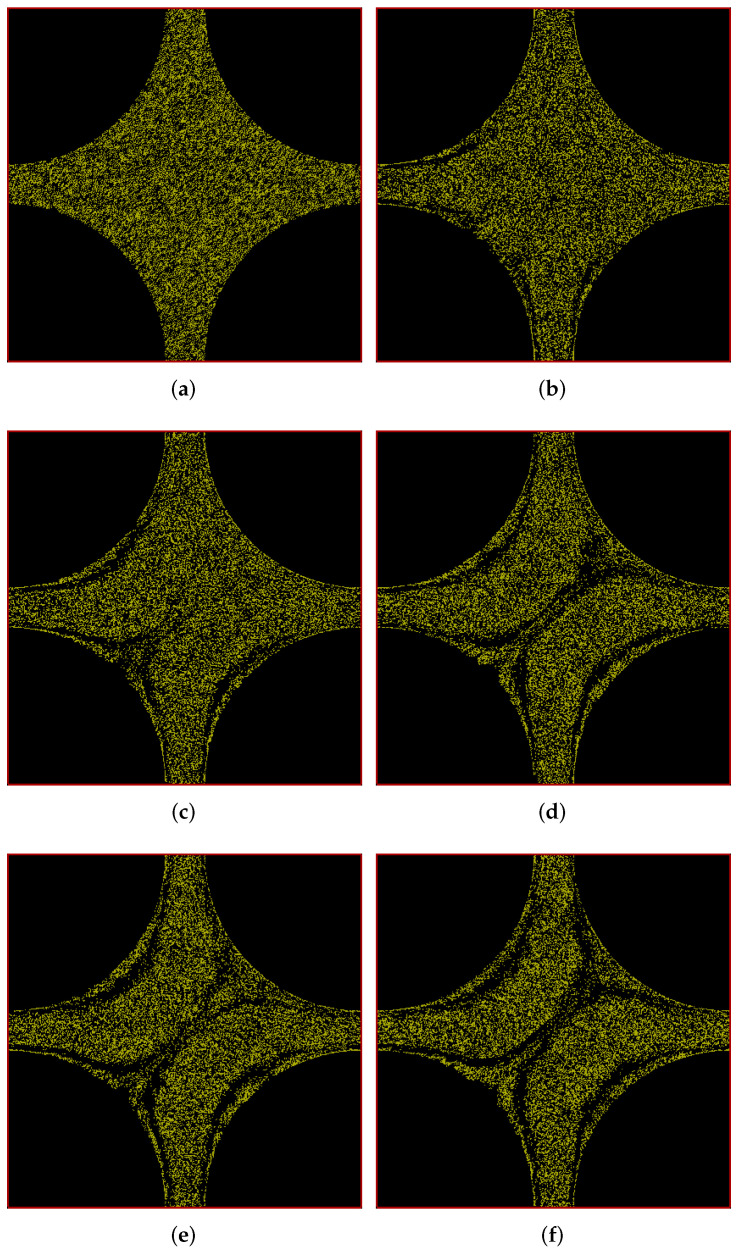
Snapshots of simulation (in porous-media-view) at different times showing development of polymer positions over time for flow through a cylindrical porous medium with pressure applied along the positive *x*-*y* diagonal. (**a**) t = 0 s; (**b**) t = 25 s; (**c**) t = 50 s; (**d**) t = 100 s; (**e**) t = 150 s; (**f**) t = 200 s.

In Figure 4, we show the positions of polymers at different times in our simulation box in porous-media-view. Here too, there is a difference between the downstream and upstream polymer distribution during the evolution of the flow, however it is less significant at the steady state. The expected mirror symmetry in the figure’s diagonal is clearly visible. In order to understand the geometry of the streaks, we take a look at the velocity profiles.

**Figure 5:**
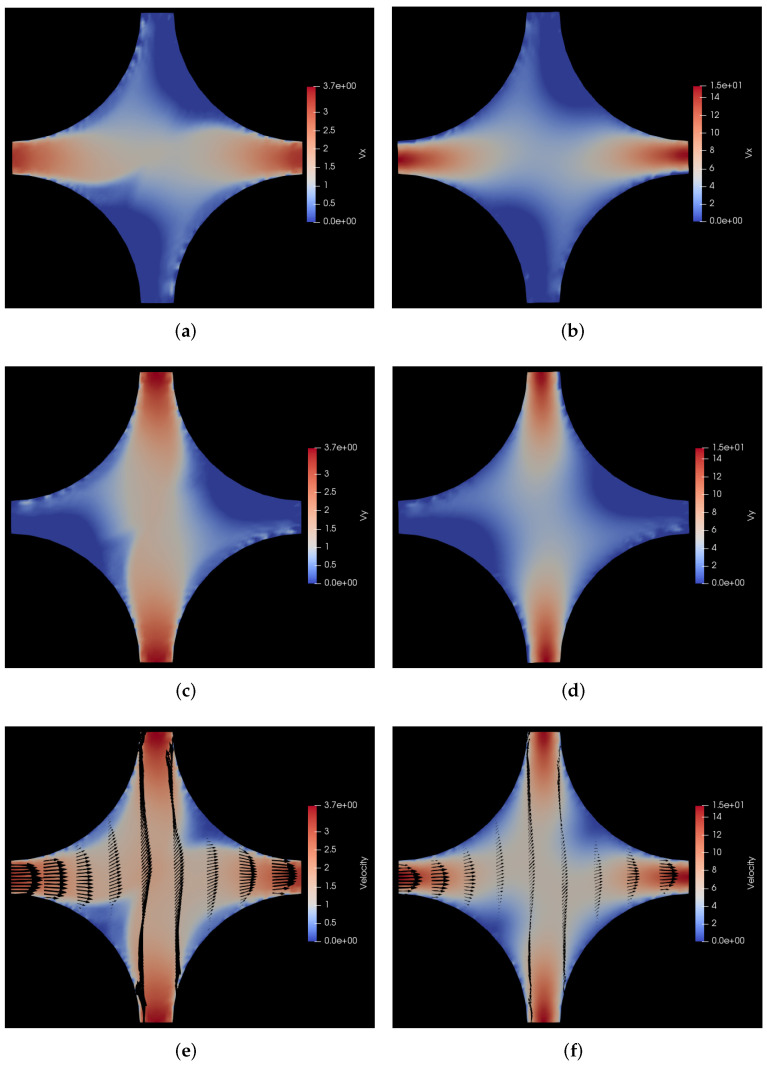
Comparison of velocity profiles for flow of polymer solution with the flow of solvent through a cylindrical porous medium with pressure applied along the positive *x*-*y* diagonal. (**a**) $v_{x}$ for flow of polymer solution. Here, $v_{x}$ varies from 0 to 3.7 σ/s, i.e., 0 to 18.5 μm/s; (**b**) $v_{x}$ for flow of solvent. Here, $v_{x}$ varies from 0 to 15 σ/s, i.e., 0 to 75 μm/s; (**c**) $v_{y}$ for flow of polymer solution. Here, $v_{y}$ varies from 0 to 3.7 σ/s, i.e., 0 to 18.5 μm/s; (**d**) $v_{y}$ for flow of solvent. Here, $v_{y}$ varies from 0 to 15 σ/s, i.e., 0 to 75 μm/s; (**e**) *v* for flow of polymer solution. Here, *v* varies from 0 to 3.7 σ/s, i.e., 0 to 18.5 μm/s; (**f**) *v* for flow of solvent. Here, *v* varies from 0 to 15 σ/s, i.e., 0 to 75 μm/s.

In Figure 5, we show these velocity profiles both for the polymer solution *vs* and the Newtonian solvent. As in the previous case with the forcing along the *x* direction, the streaks in the polymer density plots occur where the velocity gradients are largest. This is somewhat similar to local shear banding with large density coupling demonstrated in other works in the literature [62]. This is also clear from Figure 5e,f. The top and bottom parts of these plots are not very informative since all velocity vectors are parallel, but the situation in these parts of the plot can be inspected by rotating the plots by 90 degrees. Besides the characteristic flattening of the shear thinning polymer solution flow through the channels as compared to the parabolic Poiseuille flow of the Newtonian solvent, there is another interesting aspect that can be observed by comparing the last two sub-figures, i.e., Figure 5e,f.

There is not just a quantitative but also a qualitative difference in the velocity profile of the polymer solution vis-a-vis the fluid velocity profile. Upon close observation of these two sub-figures, one can see that the shape of the two stagnant regions in the bottom left and top right of the polymer solution flow shown in Figure 5e is sharper (more pointed) as compared to the solvent flow in Figure 5f. This is clearly correlated with the polymer distribution that can be seen in Figure 4f. It can be seen that the polymers concentration is clearly reduced in the regions of high shear separating the primary flow from the stagnant regions and the resulting polymer concentration then affects the flow profile too because the resultant flow profile is qualitatively different from the solvent flow. This is a good illustration of the two-way coupling between the polymers and the background fluid, which is a key feature of our HCBD model. For a more clear visualization of the evolution of polymer positions, please refer to the animations of the simulations provided in the supplementary material.

### 6.2. Flow through Cuboidal Porous Media

We now introduce sharp-edged elements in the boundaries of the flowing material. An important reason for including these model simulations is to prove that they are made possible with our simulation methodology. Their practical importance is that porous media as they occur in oil reservoirs contain lots of irregular channels with sharply pointed boundary elements. As it turns out, our treatment of boundary conditions is highly successful.

Our model system consists of solid cuboidal beams with square cross-sections and their long axes oriented along the *z* direction, arranged on a square grid on the x-y plane along the cartesian axes. The length of the side of the square cross-section of each of these cuboidal beams is 71 σ, i.e., 355 microns and the perpendicular distance between the axes of the beams in *x* as well as *y* direction is 90 σ, i.e., 450 microns. The depth of the box, i.e., its length in *z* direction is 90 microns. This leads to a porous medium with a porosity of 0.38, which is the same as the porosity that we had in case of the cylindrical beams. Again as in the case of cylindrical porous media simulations, the simulation runs for the flow of polymer solutions are started with a steady state profile of the fluid and polymers are added randomly in a uniform manner throughout the box except in the solid region and then the run is continued till steady state is achieved.

#### 6.2.1. Pressure Drop in the Positive *x* Direction

We present results of flow simulations through a cuboidal porous medium, driven by an applied pressure drop in the *x* direction, which is effected in the simulation through a body force which produces an acceleration of 0.1 m/$s^{2}$.

**Figure 6:**
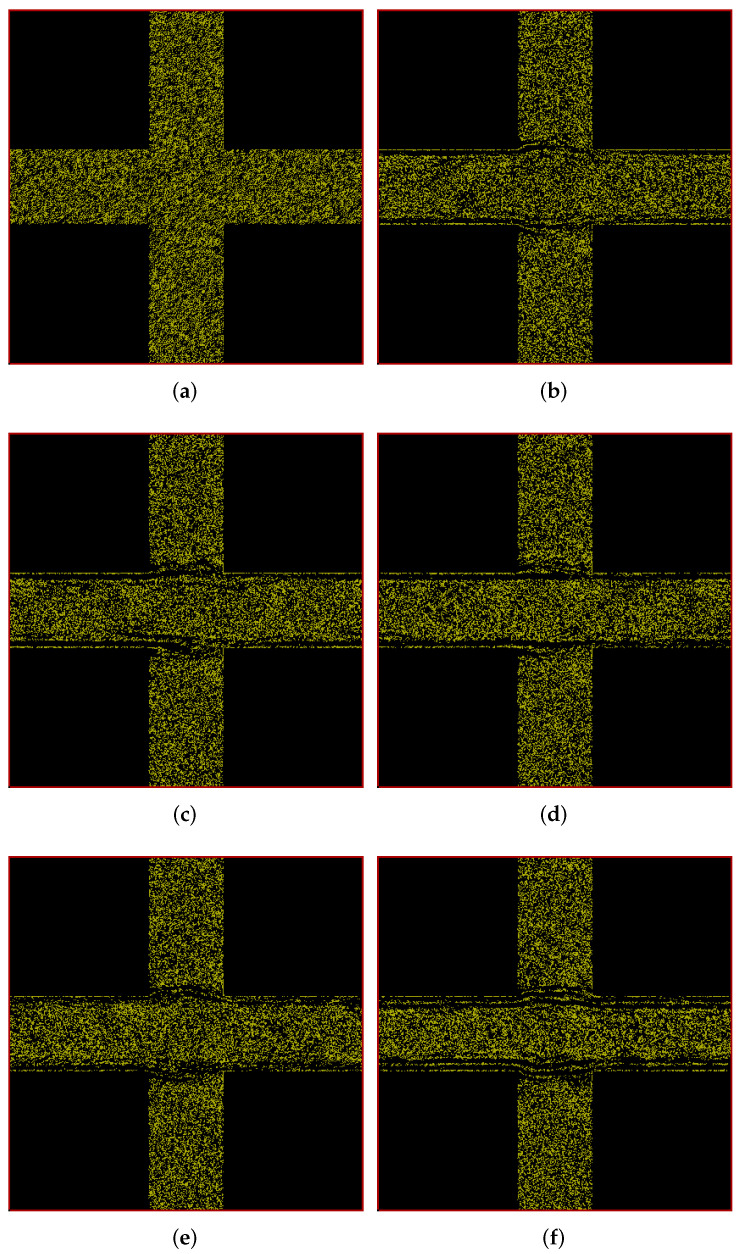
Snapshots of simulation (in porous-media-view) at different times showing development of polymer positions over time for flow through a cuboidal porous medium with pressure applied in the positive *x* direction. (**a**) t = 0 s; (**b**) t = 25 s; (**c**) t = 50 s; (**d**) t = 100 s; (**e**) t = 150 s; (**f**) t = 200 s.

In Figure 6, we show the positions of polymers at different times in our simulation box in porous-media-view. As we see in Figure 6a, the polymers are initially uniformly and randomly distributed throughout the box except the solid region. As time progresses, the polymers start forming a pattern which evolves over time and finally results in a more-or-less steady-state pattern at 200 s as shown in Figure 6f. Streaks of low polymer density appear in a similar way as with the cylindrical beams. As will be seen below, the streaks mainly appear in regions where velocity gradients are large. Interestingly, a layered structure seems to built up along the walls of the horizontal channel. The asymmetry between upstream and downstream structures is much less pronounced than with the cylindrical beams.

**Figure 7:**
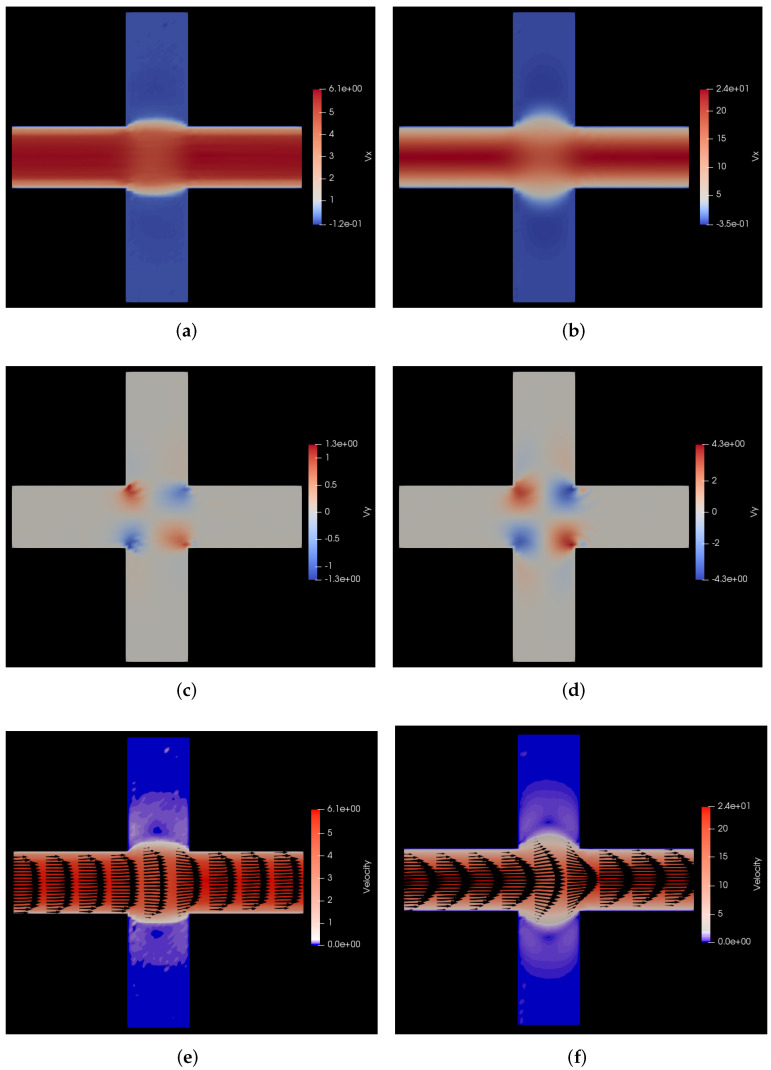
Comparison of velocity profiles for flow of polymer solution with the flow of solvent through a cuboidal porous medium with pressure applied in the positive *x* direction. (**a**) $v_{x}$ for flow of polymer solution. Here, $v_{x}$ varies from −0.12 to 6.1 σ/s, i.e., −0.6 to 30.5 μm/s; (**b**) $v_{x}$ for flow of solvent. Here, $v_{x}$ varies from −0.35 to 24 σ/s, i.e., −1.75 to 120 μm/s; (**c**) $v_{y}$ for flow of polymer solution. Here, $v_{y}$ varies from −1.3 to 1.3 σ/s, i.e., −6.5 to 6.5 μm/s; (**d**) $v_{y}$ for flow of solvent. Here, $v_{y}$ varies from −4.3 to 4.3 σ/s, i.e., −21.5 to 21.5 μm/s; (**e**) v for flow of polymer solution. Here, *v* varies from 0 to 6.1 σ/s, i.e., 0 to 30.5 μm/s; (**f**) v for flow of solvent. Here, *v* varies from 0 to 24 σ/s, i.e., 0 to 120 μm/s.

In Figure 7, we show the comparison of the steady state velocity profiles of the flow of polymer solution with those of the flow of the solvent through the same medium for the same pressure drop. On the left hand side panels of this figure, we show the polymer solution flow profile and on the right hand side panels, we show the solvent flow profile. For this flow geometry, we see interesting secondary flows induced near the cross section area of the two channels. Because of the restrictive size of the channels, the primary flow in the *x* direction does not gradually dissipate in the *y* direction as in the analogous case with cylindrical beams, but rather leads to the occurrence of two counter-rotating vortices, one above and one below the primary horizontal flow. These vortices are clearly visible as purple lobes in the vertical columns in Figure 7e,f. The counter-rotating character of the two vortices is evident from the different signatures of the flow in *y* direction above and below the horizontal mid plane in Figure 7a,b. For a more clear visualization of the counter-rotating vortices, please refer to the animations of the simulations provided in the supplementary material. Furthermore, in these two subfigures, the characteristic flattening of the velocity profile in the channels is clearly visible for the polymer solution flow vis-a-vis the parabolic Poiseuille flow profile for the Newtonian solvent flow.

#### 6.2.2. Pressure Drop along the Positive *x*-*y* Diagonal

**Figure 8:**
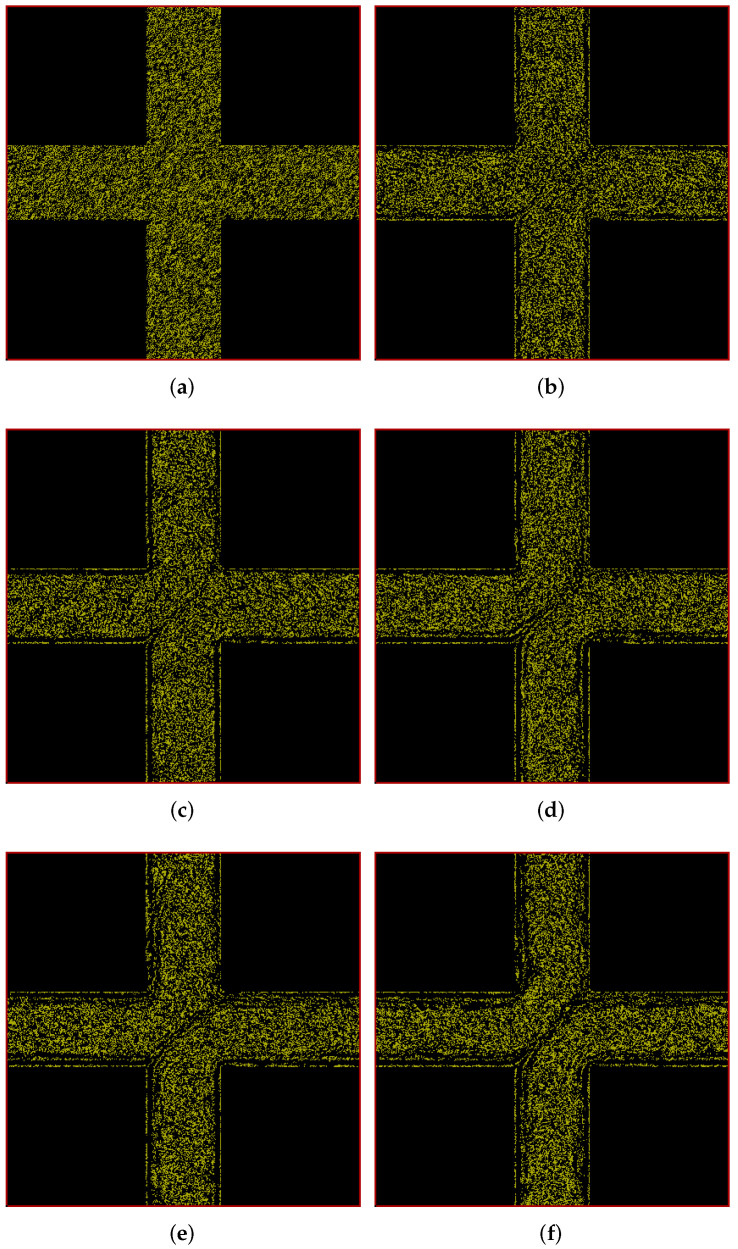
Snapshots of simulation (in porous-media-view) at different times showing development of polymer positions over time for flow through a cuboidal porous medium with pressure applied along the positive *x*-*y* diagonal. (**a**) t = 0 s; (**b**) t = 25 s; (**c**) t = 50 s; (**d**) t = 100 s; (**e**) t = 150 s; (**f**) t = 200 s.

**Figure 9:**
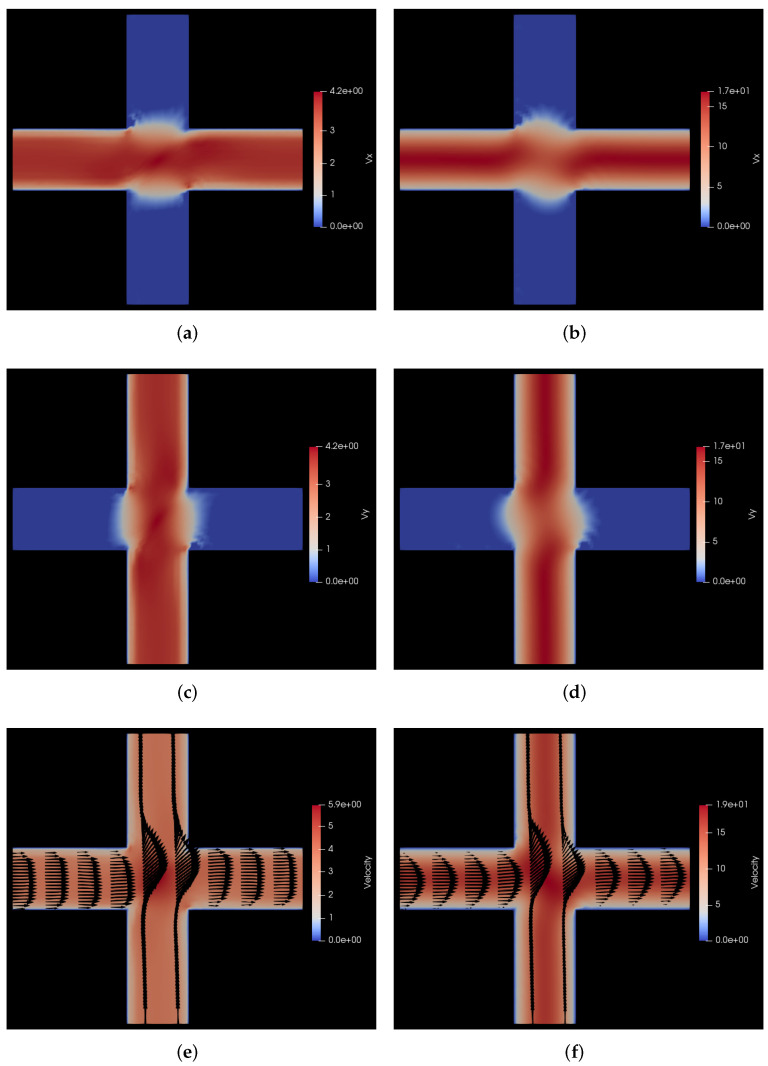
Comparison of velocity profiles for flow of polymer solution with the flow of solvent through a cuboidal porous medium with pressure applied along the positive *x*-*y* diagonal. (**a**) $v_{x}$ for flow of polymer solution. Here, $v_{x}$ varies from 0 to 4.2 σ/s, i.e., 0 to 21 μm/s; (**b**) $v_{x}$ for flow of solvent. Here, $v_{x}$ varies from 0 to 17 σ/s, i.e., 0 to 85 μm/s; (**c**) $v_{y}$ for flow of polymer solution. Here, $v_{y}$ varies from 0 to 4.2 σ/s, i.e., 0 to 21 μm/s; (**d**) $v_{y}$ for flow of solvent. Here, $v_{y}$ varies from 0 to 17 σ/s, i.e., 0 to 85 μm/s; (**e**) *v* for flow of polymer solution. Here, *v* varies from 0 to 5.9 σ/s, i.e., 0 to 29.5 μm/s; (**f**) *v* for flow of solvent Here, *v* varies from 0 to 19 σ/s, i.e., 0 to 95 μm/s.

Finally, we present results of flow simulations through our cuboidal porous medium, driven by an applied pressure drop in the diagonal direction 45 degrees to the positive *x* and *y* directions. As in the previous cases, the pressure drop is imposed in the simulation through body forces along the positive *x* and *y* directions, which together produces an acceleration of 0.1 m/$s^{2}$ along the diagonal direction. In Figure 8, we show the positions of polymers at different times in our simulation box in porous-media-view. Hardly any difference between upstream and downstream regions is visible. The low density streak along the diagonal in the cross section of the two channels is relatively weak. Again, at late times, layered structures appear near the walls. This goes along with rather large velocity gradients near the walls as seen in Figure 9.

In Figure 9, we show the comparison between the velocity profiles for our model polymer solution vs. the Newtonian flow of the solvent. It can be observed from the last two sub-figures of the above figure, i.e., Figure 9e,f that there is a characteristic flattening of the polymer solution flow through the channels as compared to the parabolic Poiseuille flow of the Newtonian solvent. Furthermore, as we expect by now, there is also a qualitative difference between the velocity profile of the polymer solution and that of the fluid, which in this case is more evident from the comparison of the velocity components, i.e., comparison of the $v_{x}$ heat maps in Figure 9a,b and comparison of the $v_{y}$ heat maps in Figure 9c,d, respectively. This can be correlated with the polymer concentration at steady state in Figure 8f. As the polymers at the intersection of the two primary flows get sheared, a depletion occurs in this zone due to the transient forces of the polymers, which then affects the fluid flow profile. For a more clear visualization of the evolution of polymer positions, please refer to the animations of the simulations provided in the supplementary material.

## 7. Conclusions and Scope for Further Research

We have proven the feasibility of large scale particle based simulations of polymer solutions through model porous media. A particle based model was chosen because of its ability to reproduce density and velocity fluctuations on a wide range of time and length scales. In order to reproduce memory in the friction forces acting on the coarse-grained degrees of freedom, originating from very slow relaxation processes in the eliminated degrees of freedom that are not captured by the conservative potential of mean force, we included so-called transient forces in the coarse model, depending on slowly developing structural parameters. Furthermore, the polymers which were represented as Finitely Extensible Non-Linear Elastic (FENE) dumbbells obeying Brownian dynamics were coupled to the SPH fluid particles in a momentum conserving way using our Hydrodynamically Coupled Brownian Dynamics (HCBD) technique, thus guaranteeing correct hydrodynamic interactions. With all these physical aspects correctly represented in our model, it turned out that large scale simulations of tens of thousands of particles could easily be run, even in geometries that pose severe difficulties to constitutive model simulations.

The model porous media were made of periodic arrays of (1) cylindrical beams with circular cross-sections and (2) cuboidal beams with square cross-sections. The axes of the beams were perpendicular to a plane, on which they are arranged with a square pitch. Two extreme angles of approach were studied in both cases. In all cases, it was easy to impose no-slip boundary conditions at the boundaries of the systems using embedded particles inside the solid regions.

We have compared our results of polymer solution flows with those of Newtonian flows through the same geometries, modeled by simple SPH simulations. We observed that there are significant quantitative and qualitative differences between the Newtonian flow of the solvent and the non-Newtonian flow of our model polymer solution through the same porous media for the same applied pressure drop. The addition of the polymers to the solvent increases the viscosity of the solvent, which leads to a reduction in the velocity of the flow of the polymer solution through the porous media. Furthermore, this increased viscosity of the polymer solution is not constant but rather shear dependent. In this paper, we see how this leads to a flatter velocity profile of the shear-thinning polymer solution in the channels as compared to the parabolic Poiseuille flow profile of the Newtonian solvent.

As scope for future research, we envisage that a hybrid model may be constructed where our technique could be used to feed information from the scale of pore throats to larger scale Computational Fluid Dynamics (CFD) simulations of oil reservoirs. In this initial study, we did not model the oil phase but rather focused on the single phase flow of polymer solution through the porous media, as that in itself is a complex subject. In principle, it should not be very difficult to add another Newtonian oil phase to our non-Newtonian polymer solution simulation but it will still require tuning the interaction between the aqueous and the oil phase in order to produce the proper thermodynamic interaction between the two phases.

## Figures and Tables

**Table 1:** Summary of system parameters.

System Parameter	Symbol	Value	Unit
Solute length scale	σ	5.0	μm
Friction coefficient	ξ	1.0 × $10^{-7}$	kg/s
Number of Kuhn segments	p	300,000	-
Concentration of polymers	*C*	2.5	C *
Maximum number density of polymers	$n_{max}^{p}$	1.0 × $10^{4}$	C *
Number of polymers	$N_{p}$	33,062	-
Flory Huggins interaction parameter	χ	0.5	-
Strength of polymer interactions	α	500	$k_{B}T$
Relaxation time	τ	1.0	s
Spring constant	*k*	50	$k_{B}T/\sigma^{2}$
Solvent length scale	*h*	10.0	μm
Resolution of fluid	${\bar{n}}^{f}$	1.9099	particles/$h^{3}$
Number of fluid blobs	$N_{f}$	13,228	-
Density of fluid	ρ	1000	kg/$m^{3}$
Viscosity of fluid	η	1.0	mPa·s
Pressure coefficient	$P_{0}$	0.13	Pa
Time step	dt	10.0	μs
Temperature	*T*	300	K

## Supplementary Materials

The following supporting information can be downloaded at: https://www.mdpi.com/article/10.3390/polym1010000/s1: Vedio S1. Animation showing development of polymer positions over time from the simulation of the polymer solution flowing through a cylindrical porous media with pressure applied in the positive *x* direction. Vedio S2. Animation showing development of polymer positions over time from the simulation of the polymer solution flowing through a cylindrical porous media with pressure applied along the positive *x*-*y* diagonal. Vedio S3. Animation showing development of polymer positions over time from the simulation of the polymer solution flowing through a cuboidal porous media with pressure applied in the positive *x* direction. Vedio S4. Animation showing development of polymer positions over time from the simulation of the polymer solution flowing through a cuboidal porous media with pressure applied along the positive *x*-*y* diagonal.
