# Supplementary figures and images for: Large Scale Hydrodynamically Coupled Brownian Dynamics Simulations of Polymer Solutions Flowing through Porous Media
Source: Polymers (Basel). 2022 Mar 31;14(7):1422. doi: 10.3390/polym14071422 (PMC9003297; doi:10.3390/polym14071422)

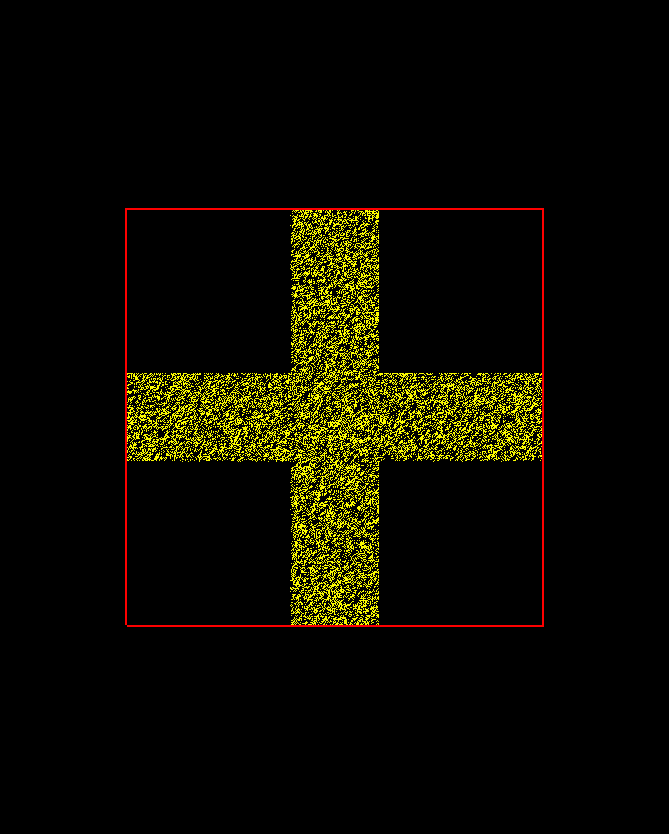

Supplement: Supplementary file 1 [file polymers-14-01422-s001.zip › Video S4. Flow in diagonal direction through periodic array of cuboidal beams.gif]

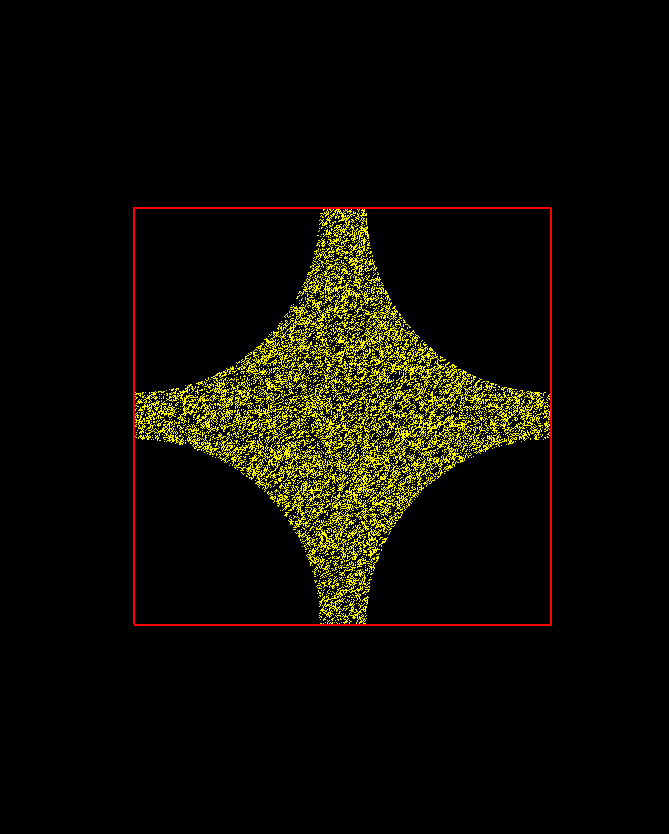

Supplement: Supplementary file 1 [file polymers-14-01422-s001.zip › Video S2. Flow in diagonal direction through periodic array of cylindrical beams.gif]

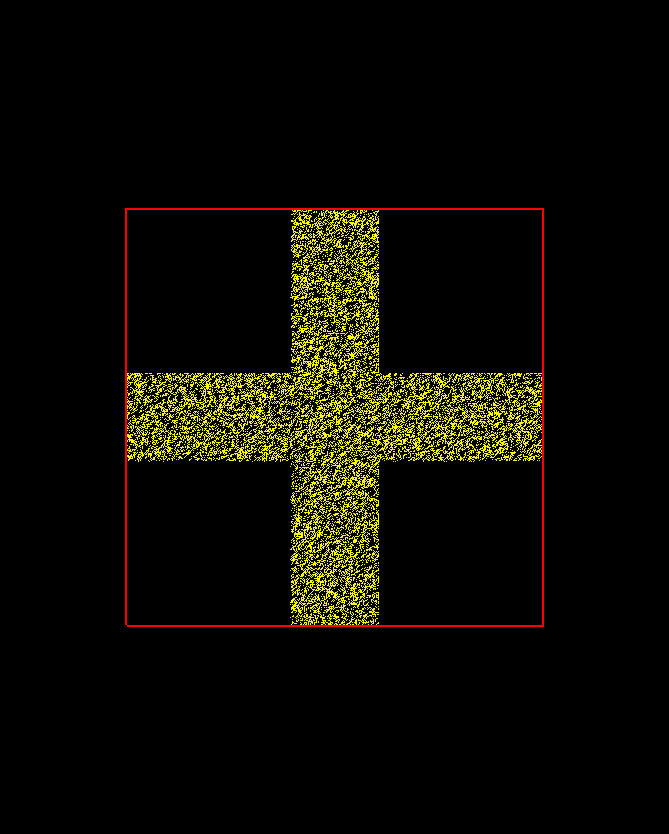

Supplement: Supplementary file 1 [file polymers-14-01422-s001.zip › Video S3. Flow in x direction through periodic array of cuboidal beams.gif]

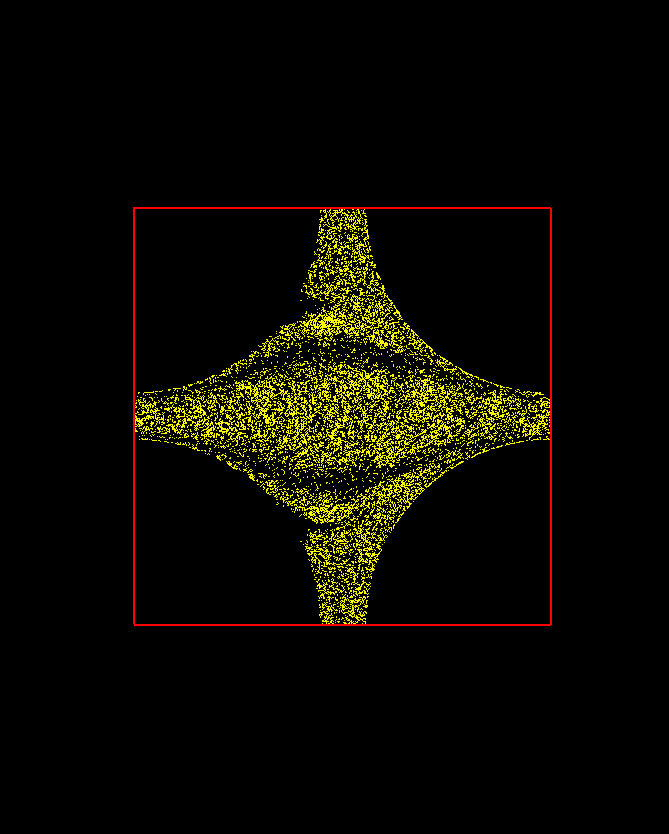

Supplement: Supplementary file 1 [file polymers-14-01422-s001.zip › Video S1. Flow in x direction through periodic array of cylindrical beams.gif]
